# Recent advances in bladder cancer stem cells (BCSCs): A descriptive review of emerging therapeutic targets

**DOI:** 10.1016/j.isci.2025.112720

**Published:** 2025-05-22

**Authors:** Shuai Li, Ke Yang, Zhaoya Jin, Pengyu Yan, Yangbin Wei, Jiawei Li, Mingquan Xu, Xinyu Guo, Qi Xing, Haojun Zhang, Quanyong Liu, Xiaofeng Yang, Chao Liu

**Affiliations:** 1 Department of Urology, First Hospital of Shanxi Medical University, Taiyuan, China; 2 First Clinical Medical College, Shanxi Medical University, Taiyuan, China; 3 Department of Thyroid, First Hospital of Shanxi Medical University, Taiyuan, China; 4 Department of Occupational Health, School of Public Health, Shanxi Medical University, Taiyuan, China

**Keywords:** Stem cells research, Cancer

## Abstract

Bladder cancer (BC) is the most common malignancy of the urinary system, characterized by high recurrence due to limited specificity and efficacy of current therapies. Bladder cancer stem cells (BCSCs), a distinct subpopulation within BC, exhibit self-renewal, tumorigenicity, and resistance to conventional treatments, playing a critical role in BC initiation, progression, metastasis, and recurrence. This study reviews the origins, biomarkers, and therapeutic potential of BCSCs, emphasizing emerging strategies targeting these cells. BCSCs can arise from urothelial stem cells or differentiated cells, with markers such as CD44, EZH2, ALDH1A1, and SOX2 enabling their identification. Aberrant activation of multiple signaling pathways, including Hedgehog, Notch, Wnt, PI3K/Akt, STAT3, and Hippo-YAP, drives BCSC function and therapy resistance. Targeting these pathways and markers offers promising therapeutic approaches to enhance efficacy and reduce recurrence. Understanding BCSC biology provides insights into improving treatment outcomes and advancing BC management strategies.

## Graphical abstract

**Figure:**
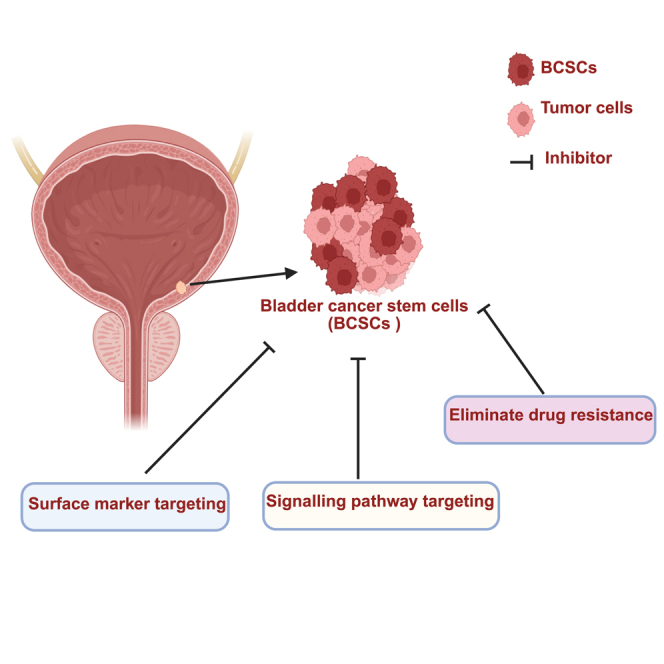

## Introduction

Bladder cancer (BC) originates in the bladder mucosa and is the tenth most common cancer worldwide. In 2022, there were 614,298 new diagnoses and 220,596 deaths due to BC.^1^ The incidence is approximately 3.3 times higher in males than in females. Smoking remains the primary risk factor, contributing to about 50% of BC cases.^2^ Urothelial carcinoma (UC) accounts for 90% of BC cases, and it is associated with a high incidence rate and frequent postoperative recurrence. Squamous cell carcinoma and adenocarcinoma each account for 2%–3%. BC can be classified into non-muscle invasive bladder cancer (NMIBC) and muscle-invasive bladder cancer (MIBC), based on tumor infiltration depth and staging. NMIBC, which includes stages Ta, T1, and Tis, comprises 70%–75% of cases and is characterized by a relatively favorable prognosis but a high recurrence rate (31%–78%). In contrast, MIBC, which includes stages T2–T4, accounts for 25%–30% of cases and is associated with a high metastatic rate and poor prognosis.^3^

Emerging evidence suggests that cancer stem cells (CSCs), a subset of self-renewing cells, drive cancer progression. CSCs act as the “seeds” of the tumor, while non-tumorigenic, differentiated cells form the “bulk” of the tumor. This hierarchical organization implies that eradicating CSCs is crucial for long-term remission and preventing recurrence, as CSCs retain regenerative potential and can drive tumor regeneration even after initial treatment-induced regression. In bladder cancer, CSCs may contribute to the high recurrence rate, tumor heterogeneity, and other complex biological behaviors. The heterogeneity of CSCs poses a significant challenge in developing targeted therapies.

## Origin and development of bladder cancer stem cells

CSCs are a small subset of tumor cells that possess stem cell properties. CSCs exhibit robust self-renewal and tumorigenic capacity, enabling them to initiate tumors in low numbers, adapt to diverse microenvironments, and differentiate into heterogeneous cancer cell phenotypes to sustain their pool; moreover, CSCs display enhanced proliferative, invasive, and migratory activities, as well as resistance to conventional therapies.^4^^,^^5^ The dual-track model proposes distinct pathways for the development of papillary and non-papillary invasive tumors. Studies have shown that low-grade tumors (which rarely progress to muscle-invasive bladder cancer) and MIBC (which are typically high-grade) originate from different cellular layers. Histological variations further complicate this model. Most low-grade tumors are papillary in nature and arise from genetic alterations in the urothelium that promote a hyperplastic phenotype. These tumors are thought to originate from the intermediate cell layer. In contrast, high-grade NMIBC develops through distinct cellular and molecular pathways, including early hyperplasia followed by severe dysplasia. Tis is believed to result exclusively from dysplasia. Both high-grade NMIBC and Tis exhibit high invasive and metastatic potential and may progress to MIBC, which originates from KRT5+ stem cells in the basal layer, with Tis serving as its precursor lesion. Mutations in genes on chromosome 9p (e.g., CDKN2A/p16INK4a), 9q (e.g., PTCH1, TSC1), STAG2, KDM6A, FGFR3, RAS, and PI3KCA characterize low-grade papillary tumors and a hyperproliferative phenotype. Conversely, mutations in TP53, MDM2, PTEN, and genomic instability are prevalent in high-grade tumors, especially MIBC.^6^

**Figure 1:**
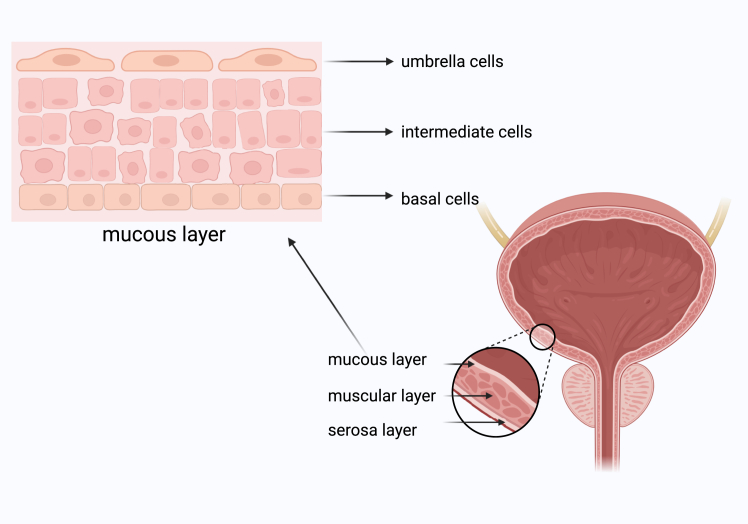
Location of urothelial cells The structure of the urothelium in the urinary bladder, highlighting umbrella cells on the luminal surface, intermediate cells, and basal stem cells. Figure created using BioRender.com.

Carcinoma of the urinary bladder originates from the urothelium, a transitional epithelium that lines the inner wall of the bladder. This multilayered epithelial structure includes fully differentiated umbrella cells on the luminal surface. Umbrella cells, which are terminally differentiated urothelial cells, are directly exposed to the bladder lumen and form a protective barrier. Under normal conditions, these cells exhibit an exceptionally slow self-renewal rate, which can extend up to 6 months.^7^ Beneath the umbrella cells are intermediate cells with limited proliferative potential, and at the base are basal cells (Figure 1). It is widely accepted that urothelial differentiation originates from stem cells residing in the basal layer. Urothelial injury, coupled with growth-stimulating signals from stromal cells (fibroblasts in the lamina propria), activates these stem cells. This process generates proliferative urothelial cells that differentiate into intermediate cells and, ultimately, umbrella cells, marking terminal differentiation.^8^

Basal cells are capable of forming bladder-like organoids from single cells *in vitro*. When genetically labeled *in vivo*, these cells can regenerate all other urothelial cell types following injury, demonstrating sustained regenerative activity over time.^9^ These characteristics suggest that basal cells may serve as bladder epithelial stem cells (BESCs). To explore the relationship between bladder cancer (BC) and BESCs, Shin et al. used Sonic hedgehog (SHH) as a marker to label basal cells and their progeny in mice. Initially, bladder intermediate and umbrella cells were unlabeled; however, all carcinoma *in situ* (CIS) and MIBC cells were eventually labeled. These results indicate that CIS and MIBC exclusively originate from basal cells expressing Sonic hedgehog (Hh) *in vivo*, confirming their derivation from BESCs. This finding supports the hypothesis that bladder cancer stem cells (BCSCs) arise from basal cells and suggests that BCSCs may be derived from BESCs. This perspective aligns with the concept that CSCs are generated from normal tissue stem cells that acquire mutations, allowing for abnormal self-renewal.^10^

Current evidence suggests that mutations or chromosomal rearrangements in progenitor cells, differentiated cells, and stem cells can contribute to the malignant transformation of CSCs.^11^ This transformation may result from the activation of oncogenic pathways independent of the cell of origin. While stem cells are the most likely source of CSCs, the possibility that more differentiated epithelial cells may also give rise to CSCs cannot be ruled out. Yang et al.^12^ performed single-cell exome sequencing on 59 cells, including BCSCs, bladder cancer non-stem cells (BCNSCs), BESCs, and bladder epithelial non-stem cells (BENSCs). Phylogenetic analysis revealed that these cells originated from either BESCs or BCNSCs. The study identified 21 driver mutation genes in BCSCs, including combined mutations in GPRC5A and MLL2, which were shown to promote the transformation of BCNSCs into BCSCs.^12^

## Molecular markers of bladder cancer stem cells

In recent years, the isolation and molecular characterization of BCSCs have gained significant attention. A deeper understanding of the biological properties of BCSCs is crucial for their effective isolation, making the identification of tumor stem cell-specific biomarkers particularly important. Markers such as CD44, ALDH1A1, COX2, SOX2, CD133, EZH2, CD47, and 67LR are considered indicative of BCSCs. Several CSC surface markers have been associated with the initiation, progression, invasiveness, maintenance of stemness, metastasis, and recurrence of bladder cancer. Identifying prognostic markers with substantial clinical impact is key to uncovering therapeutic targets for BCSCs, thereby facilitating more effective treatment strategies for bladder cancer.^13^^,^^14^

### CD44

The cell adhesion molecule CD44 is a multifunctional class I transmembrane glycoprotein that plays a crucial role in migration and cell signaling.^15^ CD44 integrates environmental and cellular signals, acting as a key regulatory factor in reactive oxygen species (ROS) metabolism in CSCs and as an important regulator of epithelial-mesenchymal transition (EMT).^16^ In BCSCs, CD44 expression is associated with tumor self-renewal, tumorigenesis, adhesion, invasion, and metastasis. Among its isoforms, the variant isoform 6 (CD44v6) is one of the most commonly used markers for CSCs. Due to the complexity of CSC markers, BCSCs can also be isolated using combinations of two or more markers. For instance, Li et al.^17^ used a combination of BCMab1 and CD44 monoclonal antibodies to isolate the BCMab1+/CD44+ subset in bladder cancer, which exhibited enhanced stem cell-like properties, including proliferation and self-renewal.

CD44 binds to hyaluronic acid, a major extracellular matrix component produced by both stromal and tumor cells.^18^^,^^19^ The interaction between hyaluronic acid and CD44 induces conformational changes that activate multiple signaling pathways, promoting cell proliferation, adhesion, migration, and invasion.^20^ For example, CD44 serves as a receptor for hyaluronic acid, which activates the Wnt/β-catenin signaling pathway. Inhibiting the interaction between CD44 and hyaluronic acid reduces tumor cell motility.^21^ Inhibiting CD44 expression significantly reduces tumor clonogenicity and tumorigenic potential. Moreover, suppression of hyaluronan synthase 1, CD44v3, CD44v6, and CD44s has been shown to inhibit the growth, invasion, and angiogenesis of bladder cancer.^22^

Chan et al. demonstrated that CD44^+^ tumor cells have tumorigenic potential 10–200 times greater than CD44^−^ tumor cells in immunodeficient mice. Analysis of tissue samples from 300 bladder cancer patients revealed that CD44^+^ cells made up approximately 40% of all tumor cells. In bladder tissue, CK20 is a marker for highly differentiated urothelial cells, while CK5 is expressed in poorly differentiated basal cells. As such, CK5 and CK20 are considered indicators of cellular differentiation status. Chan et al.^23^ identified that CD44+CK5+CK20- tumor cells retained the ability to continuously generate CSCs upon serial transplantation. In contrast, CD44-CK5-CK20+ tumor cells failed to form tumors upon serial transplantation, indicating limited capacity for renewal and/or proliferation. These findings confirm that CD44^+^ tumor cells possess self-renewal ability, and CD44+CK5+CK20- BCSCs are associated with poor prognosis in bladder cancer patients. This suggests that the diverse pathological states of bladder cancer may originate from bladder cells at varying levels of differentiation.

### ALDH1A1

Aldehyde dehydrogenase (ALDH) plays a crucial role in intracellular processes, including aldehyde detoxification and retinoic acid (RA) synthesis. ALDH activity is linked to drug resistance, a characteristic often associated with CSCs. In BCSCs, ALDH1A1 is highly expressed, and its level is directly proportional to BCSC proliferation. ALDH1A1 regulates the expression of tubulin beta-3 (TUBB3) through the RA pathway. A study using 3D cell cultures derived from patient bladder cancer cells found significant ALDH1A1 expression, a marker for stem cells. Inhibition of ALDH1A1 suppressed both proliferation and spheroid formation, and the addition of RA partially restored proliferation in ALDH1A1 knockdown cells. The researchers also showed that knocking down TUBB3, a target of the retinoic acid receptor alpha (RARα), similarly inhibited proliferation. Furthermore, ALDH inhibitors such as disulfiram (DSF) and diethylaminobenzaldehyde (DEAB) reduced TUBB3 expression. Clinical survival data suggest that TUBB3 expression correlates with poor prognosis in bladder cancer patients, indicating that ALDH1A1 and TUBB3 may serve as promising therapeutic targets.^24^

In both *in vitro* and *in vivo* xenotransplantation experiments, ALDH1A1+ cells exhibited significantly higher tumorigenic and clonogenic potential than ALDH1A1-cells. Moreover, BCSC properties were markedly reduced following shRNA-mediated knockdown of the ALDH1A1 gene. Keymoosi et al.^25^ identified the ALDH1A1+ population as a subset of CD44^+^ bladder cancer cells, which may represent a more primitive group of BCSCs. As a result, ALDH1A1 is considered a potential prognostic marker for identifying and treating high-grade UC.

Miyata et al.^26^ performed HLA ligandome analysis and cap analysis of gene expression on ALDH-high clone cells, revealing unique antigenic peptides. They identified a GRIK2 (glutamate receptor, ionotropic, kainate 2)-derived antigenic peptide (LMYDAVHVV) specifically expressed by CSCs. A GRIK2 peptide-specific cytotoxic T lymphocyte (CTL) clone was able to recognize GRIK2-overexpressing UM-UC-3 cells and ALDH-high clone cells, suggesting that GRIK2 peptides could serve as novel immunotherapeutic targets for bladder cancer CSCs/CICs.

### COX2

COX2 is expressed in bladder cancer (BC) cells but not in normal urothelium. Chemotherapy-induced apoptotic cells release PGE2, which promotes the proliferation of CSCs and tumor regeneration during chemotherapy cycles. The COX2/PGE2 signaling pathway induces methylation of the let-7 promoter in BC cells, leading to its downregulation and subsequent upregulation of SOX2. SOX2, a key transcription factor, promotes pluripotency and self-renewal in embryonic stem cells and induces the formation of induced pluripotent stem cells. Activation of the COX2/PGE2 pathway also contributes to acquired resistance of basal BC cells to EGFR inhibitors.^27^ Metformin has been shown to inhibit BC progression by suppressing CSC proliferation via the COX2/PGE2/STAT3 axis. Pan et al.^28^ first reported that metformin blocks the progression from precancerous lesions to invasive tumors by directly inhibiting the STAT3 pathway and COX2 activation. In animal and cell models, cells expressing cytokeratin 14 and Oct3/4+ were found to be sensitive to metformin’s inhibitory effects on the BCSC population. These findings suggest that metformin suppresses BC development by targeting the COX2/PGE2/STAT3 axis. A phase II clinical trial (NCT03379909) involving 49 patients with low-grade, MIBC is currently underway to assess the overall response to 3 months of oral metformin treatment.^29^ If successful, metformin may become a novel therapeutic option for BC. Additionally, PGE2-neutralizing antibodies and the COX2 inhibitor celecoxib effectively suppress PGE2 production, preventing CSC regeneration during chemotherapy intervals and significantly reducing chemoresistance progression.^28^

### Sox2

All multicellular animals possess SRY-related high mobility group (HMG) box (Sox) proteins, which are transcriptionally active and play essential roles in various developmental processes. The transcription factor Sox2, a member of the Sox family, has long been used as a marker for identifying CSCs. While Sox2 is minimally expressed in normal bladder epithelium, its expression is significantly elevated in bladder cancer. Sox2+ cells isolated from bladder cancer tissues demonstrate a greater ability to regenerate tumors compared to Sox2-cells.^30^ Therefore, Sox2 serves not only as a diagnostic marker but also as a prognostic factor for bladder cancer progression. BCSCs, characterized by the CD44+ALDH1+ phenotype, were isolated from SW780 and 5637 bladder cancer cell lines using flow-activated cytometric sorting. Quantitative reverse transcription-polymerase chain reaction (qRT-PCR) analysis revealed a significant upregulation of both the transcription factor SOX2 and its overlapping transcript, the long non-coding RNA SOX2OT, in BCSCs compared to non-BCSCs (BCNSCs), with expression levels approximately 100-fold higher. Functional characterization through *in vitro* assays, including sphere formation, 5-ethynyl-2′-deoxyuridine (EdU) incorporation, and colony formation assays, demonstrated that knockdown of SOX2OT attenuated the self-renewal capacity of BCSCs. Moreover, *in vivo* studies using xenograft models showed that inhibition of SOX2OT significantly suppressed tumor growth and reduced metastatic potential. These findings suggest that SOX2OT plays a critical role in maintaining the stemness and tumorigenicity of BCSCs and may serve as a potential therapeutic target for bladder cancer.^31^

### CD133

CD133, also known as Prominin-1, is a pentaspan transmembrane glycoprotein localized to the protrusions of adult stem cell membranes, where it plays a crucial role in maintaining stem cell properties by inhibiting differentiation. Initially introduced as a marker for hematopoietic stem cells, CD133 is now widely used to isolate and identify CSCs from various tumors, including BCSCs.^32^ Studies have shown that the presence of CD133 significantly influences muscle invasiveness and the development of higher-grade tumors in bladder cancer.^33^ Therefore, CD133 serves as a potential prognostic marker for poor outcomes in bladder cancer.

### Oct4

Oct4, a member of the POU domain gene family, is a critical transcription factor and multifunctional regulatory element involved in the self-renewal and differentiation of embryonic stem cells.^34^ Yaser et al.^35^ used immunohistochemistry with a polyclonal anti-Oct4 antibody to examine the distribution of Oct4 protein in bladder tumor tissues. In combination with other studies, their findings indicate that Oct4 is highly expressed in the side population cells of bladder cancer, which exhibit CSC-like properties. Therefore, Oct4 may serve as a novel molecular marker for BCSCs, providing a valuable tool for detecting and predicting the progression and metastasis of bladder cancer.

### EZH2

Wang et al.^36^ generated a comprehensive cancer cell atlas comprising 54,971 single cells. The regulatory genes of BCSCs can be categorized into two main groups. One group is primarily enriched for KDM5B, a histone H3K4me3 demethylase, which has been shown to be overexpressed in various human cancers, including bladder cancer.^37^ The other group is enriched for EZH2. Their study found that during bladder cancer recurrence, CSC subpopulations become enriched, and EZH2 expression is elevated. Further research demonstrated that EZH2 can directly suppress the expression of the NCAM1 gene through H3K27me3 modification. NCAM1, a cell adhesion protein, is downregulated in this process, which may be associated with increased invasiveness and stemness in cancer cells, thus promoting bladder cancer progression and recurrence. Due to its enzymatic activity, EZH2 represents a potential drug target.

### 67LR

BCSCs are characterized by positivity for the 67 kDa laminin receptor (67LR) and cytokeratin 17 (CK17), but are negative for carcinoembryonic antigen-related cell adhesion molecule 6 (CEACAM6). Additionally, abnormal activation of the WNT signaling pathway has been observed in BCSCs, with a significant increase in WNT factor expression in bladder cancer cells exhibiting high 67LR expression. Researchers isolated 67LR+/CK17+/CEACAM6-cells from the human bladder cancer cell line SW780 and bladder cancer samples. In mouse models, these cells demonstrated tumorigenic potential 5 to 10 times greater than that of other cells. These findings support the use of 67LR+/CK17+/CEACAM6-as biomarkers for the isolation and identification of BCSCs.^38^

Literature unanimously agrees that no single marker uniquely labels bladder CSCs. Instead, CD44 and ALDH1A1 consistently emerge as the most robust indicators of the CSC phenotype. For example, CD44 is highly expressed in muscle-invasive tumors and drives EMT, invasion, and angiogenesis, whereas ALDH1A1 marks a subpopulation with enhanced tumorigenicity and correlates with tumor progression, recurrence and poor survival.^39^ In fact, immunohistochemical studies show that ALDH1A1-positive cells often co-express CD44, and patients with CD44+ALDH1A1+ tumors have worse outcomes.^25^ These two markers also have clear clinical relevance: CD44 is a cell-surface molecule that can be targeted by therapeutic antibodies or exploited via hyaluronic acid–linked drug delivery, and elevated urinary CD44 levels have been proposed as a non-invasive prognostic indicator.^40^ Likewise, high ALDH1A1 activity is detected in aggressive bladder tumors and even in patient serum, and ALDH inhibitors can sensitize CSCs to chemotherapy. Thus, CD44 and ALDH1A1 not only underpin key stem-cell functions (self-renewal and chemoresistance) but also stratify patients: multiple studies report that ALDH1A1 expression predicts poor prognosis and that its knockdown suppresses tumorigenicity.

Other proposed markers—COX2, SOX2, CD133, OCT4, EZH2, 67LR, and CEACAM6—are less definitive. Transcription factors like SOX2 and OCT4 are certainly enriched in the stem-like compartment and associated with high-grade disease,^41^ but being intracellular they are harder to target and are also seen in normal stem cells. COX2 (via PGE2) and YAP1 cooperate to sustain CSCs, and their joint inhibition can abrogate bladder CSC expansion, yet COX2 is broadly induced by inflammation and not specific to CSCs. CD133 has been reported on some bladder CSCs, especially in non–muscle-invasive tumors, but its expression is highly variable and often overlaps with normal progenitors. Similarly, EZH2 (an epigenetic stemness regulator) is overexpressed in many bladder tumors and linked to metastasis,^42^ making it a candidate CSC-associated gene, but it is not a surface marker and is also active in proliferating non-CSC tumor cells. Markers like the 67-kDa laminin receptor (67LR) or CEACAM6 have shown CSC-like associations in other cancers, but in bladder their roles remain uncertain and need further study.

In summary, current evidence supports prioritizing CD44 and ALDH1A1 as the most biologically and clinically meaningful BCSC markers. Their expression is closely tied to stemness traits, chemoresistance, and poor patient outcomes, and they offer concrete therapeutic angles. At the same time, the field recognizes that no marker is perfect: true CSC targeting will likely require multi-marker panels and context-dependent validation to overcome heterogeneity and normal-cell overlap.

## Signaling pathways in bladder cancer stem cells and therapeutic targets

Understanding the role and regulatory mechanisms of BCSCs may contribute to the development of effective treatments and improved prognostic outcomes. Mutations in key genes within signaling pathways that normally regulate stem cell self-renewal and differentiation can disrupt these pathways or lead to their aberrant activation, ultimately resulting in tumorigenesis. The Hedgehog signaling pathway, the Hippo-YAP pathway, and EMT-related pathways such as Wnt/β-catenin, Notch, STAT3, and PI3K/AKT are all implicated in the initiation and progression of BCSCs. These signaling cascades play critical roles in regulating various biological processes in bladder cancer cells, including proliferation, differentiation, apoptosis, senescence, and self-renewal.

### Wnt signaling pathway

The Wnt signaling pathway is pivotal in regulating a variety of cellular processes, including stem cell self-renewal, differentiation, and tissue homeostasis, while also playing a significant role in cancer initiation and progression. The pathway comprises Wnt ligands, Frizzled receptors, LRP5/6 co-receptors, the destruction complex (which includes AXIN, GSK3β, and APC), and β-catenin. Upon the binding of Wnt ligands to these receptors, a cascade of complex signaling events is triggered. The Wnt pathway can be categorized into two primary branches: the canonical Wnt/β-catenin pathway and the non-canonical Wnt pathways. In bladder cancer, the canonical Wnt/β-catenin signaling pathway is frequently dysregulated. Aberrant nuclear accumulation of β-catenin is observed in high-grade and muscle-invasive bladder tumors, and is associated with upregulation of Wnt target genes such as c-myc and cyclin D1 that drive tumor progression and maintain CSC-like properties.^43^ In highly differentiated bladder cancer cells, Wnt signaling inhibitors are present, which suppress the activity of Wnt pathway components, preventing the activation of downstream molecule β-catenin. In contrast, excessive activation of the Wnt pathway can lead to the overproliferation of CSCs.^44^^,^^45^ In urothelial stem cells, single nucleotide polymorphism (SNP) genotyping of 40 genes within the Wnt/β-catenin signaling pathway has revealed variations in this pathway, which have been shown to play a role in the pathogenesis of bladder cancer.^46^

**Figure 2:**
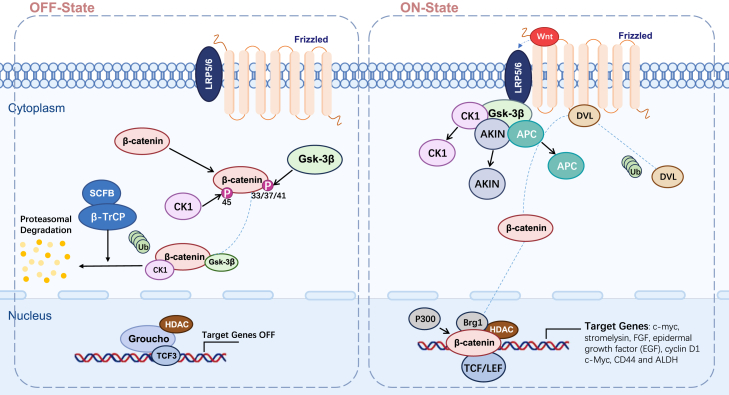
Canonical Wnt/β-catenin signaling pathway Canonical Wnt/β-catenin signaling pathway: the Wnt/β-catenin pathway regulates cell proliferation, differentiation, and survival. Inactive Wnt signaling promotes β-catenin degradation, preventing gene activation. Active Wnt signaling stabilizes β-catenin, allowing its nuclear translocation, where it binds to TCF/LEF transcription factors to activate genes like c-myc, cyclin D1, and CD44. This pathway is crucial in development, tissue homeostasis, and cancer progression.

Canonical Wnt/β-catenin Pathway: (1) Inactive Wnt signaling: In the absence of Wnt signaling, β-catenin is tagged for degradation in the cytoplasm through a series of phosphorylation events. Casein kinase 1 (CK1) first phosphorylates β-catenin at Ser45, followed by phosphorylation by glycogen synthase kinase-3β (GSK-3β) at Thr41, Ser37, and Ser33. These modifications create binding sites for the SCFb-TrCP ubiquitin ligase, leading to the ubiquitination and proteasomal degradation of β-catenin. This ensures that β-catenin remains at low levels in the cytoplasm, preventing its nuclear translocation and activation of gene transcription. (2) Active Wnt signaling: When the Wnt ligand binds to Frizzled (FZD) receptors and co-receptors such as LRP5 and LRP6, the pathway is activated. This binding triggers the interaction between FZD receptors and Disheveled (DVL) proteins. The activated DVL protein promotes the phosphorylation of LRP5 and LRP6, forming a complex with the FZD receptor. This complex recruits AXIN to the inner membrane, where it interacts with DVL and LRP5/6 to inhibit the β-catenin degradation complex (DVL/AXIN/APC/GSK-3β/CK1). This prevents the further phosphorylation and degradation of β-catenin. Stabilized β-catenin accumulates in the cytoplasm, eventually translocating into the nucleus. In the nucleus, β-catenin binds to T cell factor/lymphoid enhancer factor (TCF/LEF) family transcription factors, replacing Groucho to form a transcriptional activation complex. This complex recruits co-activators such as histone acetyltransferases, p300, and Brg1, initiating chromatin remodeling and the transcription of target genes such as c-myc, cyclin D1, and CD44^47^ (Figure 2).

Importantly, bladder cancers exhibiting resistance to chemotherapy, such as paclitaxel, often show enhanced β-catenin signaling. Overexpression of β-catenin in these cells maintains CSC-like traits, while pharmacological inhibition of β-catenin sensitizes them to treatment.^48^ Recent research has identified PPP2R2B as a novel biomarker after analyzing mitochondrial-related genes. PPP2R2B is expressed at lower levels in BC specimens, and its overexpression inhibits the Wnt/β-catenin/EMT pathway, thus suppressing BC cell proliferation and metastasis. This study confirms PPP2R2B as an anti-cancer gene involved in BC progression^49^ (Table 1). Another study found that SLC14A1-positive tumor-associated fibroblasts (irCAFs) induce a CSC-like phenotype in cancer cells by secreting WNT5A, significantly affecting prognosis.^50^ Endostatin, by blocking β-catenin function, leads to the downregulation of the Wnt/β-catenin pathway, suppressing BC cell growth and migration. It also inhibits angiogenesis.^51^ Additionally, increasing doses of Qici Sanling decoction significantly suppressed β-catenin, survivin, c-myc, and cyclin D1, thereby inhibiting bladder cancer growth^52^ (Table 2). Thymoquinone (TQ), a major bioactive compound from black seed oil (*Nigella sativa*), reverses EMT by upregulating epithelial markers (e.g., E-cadherin) and downregulating mesenchymal markers (e.g., N-cadherin and vimentin), inhibiting BC cell metastasis. TQ also inhibits the expression of β-catenin target genes, such as MYC, Axin-2, MMP7, CyclinD1, and MET, which play crucial roles in EMT and cancer progression, further suppressing BC cell invasion and metastasis.^53^

**Table 1:** Emerging therapeutic targets

Proposed therapeutic targets	Signal pathway	Impact on cancer	Reference
PPP2R2B	classic Wnt/β-catenin	PPP2R2B overexpression inhibits the proliferation and metastasis of BC cells.	Shen and Kang^49^
SLC14A1	classic Wnt/β-catenin	Secretion of WNT5A, which functions through the classical Wnt/β-catenin pathway, induces cancer cells to acquire stemness.	Ma et al.^50^
SFRP1	non-classical Wnt/β-catenin	Overexpression of SFRP1 leads to a reduction in the viability and migratory ability of bladder cancer (BC) cells, thus lowering their malignant potential.	Rogler et al.^54^
SOX4	non-classical Wnt/β-catenin	SOX4 enhances the invasiveness of BC cells by inhibiting Wnt5a in the non-classical Wnt pathway.	Moran et al.^55^
THZ1	Sonic Hedgehog	The interference with the SHH signaling pathway suppresses cancer stem cell characteristics.	Chow et al.^56^
Bromodomain-4 protein (BRD4)	Sonic Hedgehog	BRD4 positively regulates the SHH pathway, enhancing the migration and invasion of bladder cancer cells.	Liu et al.^57^
Regulator of cullins-1 (ROC1)	Sonic Hedgehog	Overexpression of ROC1 targets the fusion homologue (SUFU) inhibitor for ubiquitin-dependent degradation, promoting bladder cancer cell growth.	Wang et al.^58^
Laminins	Notch	Laminins activate the Notch pathway through the integrin α6β4/TRB3/JAG3, triggering the proliferation or migration of tumor cells in bladder cancer.	Hao et al.^59^
Circ_0008532	Notch	Circ_0008532 mediates the expression of miR-155-5p/miR-330-5p target gene MTGR1 and downstream Notch signaling, thus playing an oncogenic role in BC.	Chen et al.^60^
TEAD4	PI3K/Akt	TEAD4 activated PI3K/AKT pathway in BLCA cells.	Chi et al.^61^

**Table 2:** Emerging therapeutic drug

Proposed therapeutic drug	Signal pathway		Reference
Endostatin	classic Wnt/β-catenin		Wu et al.^51^
Qici Sanling decoction	classic Wnt/β-catenin		Gong et al.^52^
Thymoquinone	classic Wnt/β-catenin	Upregulation of epithelial markers (e.g., E-cadherin) and downregulation of mesenchymal markers (e.g., N-cadherin and vimentin) to reverse EMT and inhibit distant metastasis of BC cells.	Zhang et al.^53^
analogue CYD 6–17	non-classical Wnt/β-catenin	This treatment exerts a potent inhibitory effect on bladder cancer cells, overcomes drug resistance, and causes no significant side effects.	Zhou et al.^62^
Epigallocatechin-3-gallate (EGCG)	Hedgehog	EGCG inhibited bladder cancer tumorspheres, downregulated stem cell markers, suppressed the expression of proliferation-associated proteins in and promoted the apoptosis of bladder CSCs.	Sun et al.^63^
myrtucommulone-A	PI3K/Akt	Myrtucommulone-A (MC-A) has been found to negatively regulate the EMT process by effectively diminishing mTOR activity and modulating the PI3K/AKT signaling pathway.	Iskender et al.^64^
Pictilisib, buparlisib, pilaralisib	PI3K/Akt	These inhibitors exhibit broad anticancer activity by targeting and inhibiting multiple PI3K isoforms.	He et al.^65^
MK-2206	PI3K/Akt	MK-2206 effectively alleviated the promotional effects of UPP1 on BLCA tumor growth.	Du et al.^66^
Verteporfin	Hippo-YAP	VP inhibits bladder cancer cell proliferation by specifically blocking the YAP-TEAD complex.	Dong et al.^67^
WP1066	STAT3	WP1066 blocks STAT3 phosphorylation in bladder cancer cells, reducing cell survival and proliferation.	Tsujita et al.^68^
Pae	STAT3	Pae inhibits the proliferation of human BCa cell lines in a dose- and time-dependent manner.	Yang et al.^69^
δ-tocotrienol	STAT3	δ-Tocotrienol induces apoptosis in bladder cancer cells by reducing the levels of STAT3 protein and its transcriptional activity within the nuclear compartment.	Jiang et al.^70^

In addition to the canonical Wnt signaling pathway, the role of the non-canonical Wnt pathway in BC has also been validated. The Wnt signaling antagonist SFRP1 is a promising marker in bladder cancer. SFRP1 expression in bladder tumors is associated with lower tumor grades. Experimental results show that high promoter methylation inhibits SFRP1 expression, activating the non-canonical Wnt pathway. Overexpression of SFRP1 in tumor cells decreases BC cell viability and migration, reducing malignancy. In terms of prognosis, bladder cancer cells expressing SFRP1 show a trend toward prolonged overall survival.^54^ Sry-related HMG-box-4 (SOX4), a developmental transcription factor, is overexpressed in many bladder cancer patients. Studies show that SOX4 enhances BC cell invasiveness by inhibiting WNT5a, which acts via the non-canonical Wnt pathway. High WNT5a expression correlates with reduced BC cell invasiveness, and WNT5a expression is inversely correlated with SOX4 expression. This suggests that SOX4 may regulate WNT5a levels either directly or indirectly, and WNT5a play a protective role in preventing invasion in bladder cancer cells. SOX4 serve as a potential target for reducing BC cell invasiveness.^55^ Chen et al.^62^ identified a novel natural product analog, CYD 6–17, which inhibits bladder cancer cells by preventing XBP1 binding to the β-catenin gene promoter. This compound effectively overcomes drug resistance without significant side effects.

### Hedgehog signaling pathway

**Figure 3:**
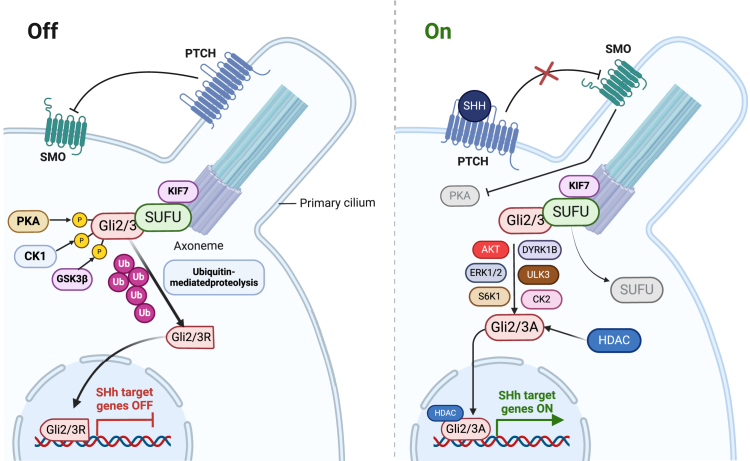
Hedgehog (Hh) signaling pathway The Hedgehog (Hh) signaling pathway regulates embryogenesis, cell proliferation, and tissue repair. SHH binds to PTCH1, activating SMO, which triggers GLI protein activation. GLI2/3 translocate to the nucleus, regulating gene transcription. This pathway is essential for development, stem cell maintenance, and cancer progression, with tight regulation through phosphorylation, ubiquitination, and acetylation processes. Figure created using BioRender.com.

The Hedgehog (Hh) signaling pathway plays a pivotal role in the regulation of embryogenesis and development. The activation of the SHH signaling pathway begins when the SHH glycoprotein binds to the Patched1 (PTCH1) receptor on the cell membrane. This binding releases the inhibitory effect of PTCH1 on Smoothened (SMO), thereby activating SMO. Once inside the primary cilium, SMO is further phosphorylated by casein kinase 1α. This phosphorylation state facilitates the recruitment of kinesin family member 3A (Kif3A) to the SMO-β-arrestin complex. Kif3A interacts with the Kinesin family member 7 (KIF7)-SUFU-GLI2/3 complex within the primary cilium, supporting the correct localization and function of GLI proteins. As SMO activation occurs, the KIF7/SUFU/GLI2/3 complex dissociates from GLI2/3. These GLI proteins then undergo a series of post-translational modifications, including phosphorylation, ubiquitination, and SUMOylation. Several protein kinases, including casein kinase II (CK2), protein kinase B (AKT), extracellular signal-regulated kinase 1/2 (ERK1/2), ribosomal protein S6 kinase 1 (S6K1), dual-specificity tyrosine phosphorylation-regulated kinase 1B (DYRK1B), and unc-51 like kinase 3 (ULK3), phosphorylate GLI2/3, promoting its transport to the nucleus. The transcriptional activity of GLI proteins is further regulated by acetylation and deacetylation. The acetylation of GLI1/2, mediated by the p300/CBP complex, prevents GLI proteins from binding to DNA and promotes their export from the nucleus. Conversely, deacetylation by histone deacetylase HDAC1 enables GLI proteins to bind to genomic DNA. HDAC1 is upregulated by GLI proteins, creating a positive feedback loop in the SHH pathway. After GLI proteins are activated and transported to the nucleus, GLI2/3 bind to DNA and regulate the transcription of target genes. These target genes, involved in embryonic development, cell proliferation, and tissue repair, are activated by GLI proteins interacting with transcription factors such as GLI1 and GLI2^71^ (Figure 3). In tumor tissue, cancer-associated fibroblasts (CAFs) are activated by paracrine stimulation of SHH signaling, which may be induced by SHH proteins released from tumor cells or CSCs.^71^^,^^72^^,^^73^ The SHH signaling target proteins secreted by CAFs not only promote angiogenesis (via VEGF-dependent mechanisms) but also support the self-renewal of CSCs, leading to their resistance to multiple chemotherapeutic agents and playing a significant role in tumor progression and relapse.^74^^,^^75^ Furthermore, recent reviews indicate that CAFs, including resident fibroblasts and mesenchymal stem cells, contribute to tumor progression by remodeling the extracellular matrix and modulating immune responses, with SHH signaling being a key factor in their activation.^74^

Bromodomain-4 protein (BRD4) is upregulated in bladder cancer tissues and cells, where it enhances the migration and invasion of cancer cells by positively regulating the sonic hedgehog (SHH) signaling pathway. Moreover, the pharmacological effects of cisplatin (DDP), a chemotherapy agent used to inhibit tumor growth *in vivo*, are reduced by the overexpression of BRD4, suggesting that BRD4 could serve as a novel therapeutic target for bladder cancer.^57^ In addition, the covalent cyclin-dependent kinase 7 (CDK7) inhibitor THZ1 inhibits cancer stemness by disrupting SHH signaling. This results in effective apoptosis induction and reduced cell viability in UC cell lines and xenograft models, including those resistant to chemotherapy, indicating THZ1’s potential as a therapeutic strategy for UC.^56^ Regulator of cullins-1 (ROC1), an essential subunit of the cullin-RING ligase (CRL) complex, is crucial for the survival and progression of bladder cancer cells. ROC1 overexpression leads to the ubiquitin-dependent degradation of SUFU, an inhibitor of Gli2, allowing Gli2 to activate the SHH pathway and promote cancer cell growth.^58^ Epigallocatechin-3-gallate (EGCG), a bioactive polyphenol from green tea, has been widely studied for its anticancer effects, including its impact on bladder cancer. Evidence indicates that EGCG’s inhibitory effects are mediated through the SHH signaling pathway, and upregulation of SHH components diminishes EGCG’s efficacy.^63^

### Notch signaling pathway

**Figure 4:**
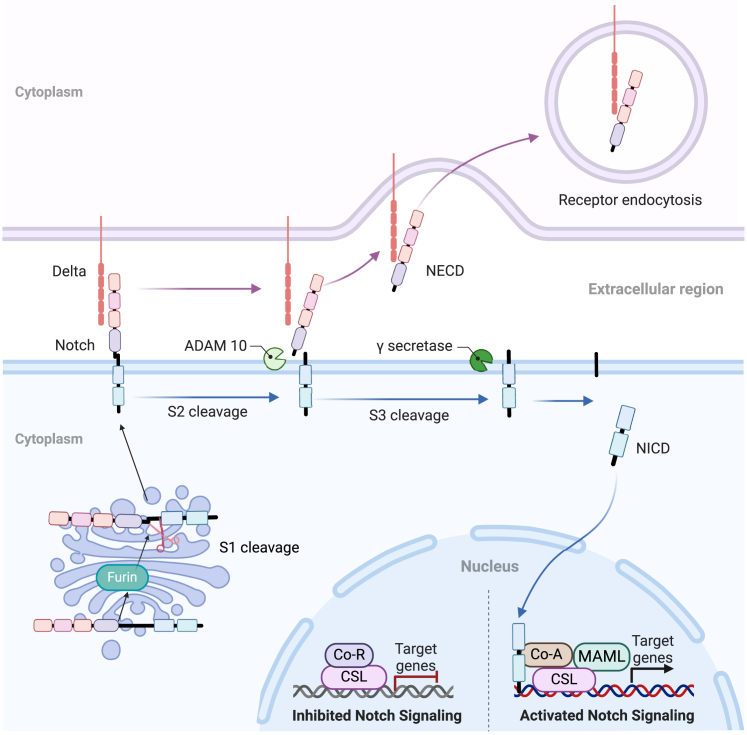
Notch signaling pathways The Notch signaling pathways, highlighting the activation, cleavage, and nuclear translocation of Notch proteins, as well as their crosstalk with other signaling pathways involved in cellular differentiation, proliferation, and tumorigenesis. Figure created using BioRender.com.

The Notch signaling pathway is highly conserved throughout evolution and plays a crucial role in various processes during normal cellular morphogenesis, including the regulation of proliferation, stem cell maintenance, and differentiation in embryonic and adult development. The Notch signaling pathway is named after the Notch receptors, This pathway involves four receptors (Notch1 to Notch4), which consist of three main regions: the Notch extracellular domain (NECD), the transmembrane domain (TMD), and the Notch intracellular domain (NICD), along with five ligands (JAGGED1, JAGGED2, DLL1, DLL3, and DLL4).^76^ High expression of NOTCH2/3 and their ligand DLL4 correlates with increased tumor cell viability and proliferation, suggesting the importance of the NOTCH2/3-DLL4 axis as a potential therapeutic target.^77^ The classical Notch pathway plays a primary physiological role in cell-cell interactions and gene transcription regulation, while the non-classical pathway involves crosstalk with other signaling pathways to activate target genes. The classical Notch pathway begins with the maturation and activation of Notch proteins. These proteins are initially transported as single-chain precursors within the endoplasmic reticulum, where their EGF-like domains undergo glycosylation. The glycosylated Notch precursor is then transported to the Golgi apparatus, where a furin-like convertase cleaves the protein at the S1 site, producing two distinct fragments: the NECD and TMD. These fragments are held together by calcium-dependent non-covalent bonds, forming a mature Notch receptor heterodimer. The mature receptor is transported to the cell surface, where it interacts with Notch ligands on adjacent cells. The Notch receptor is cleaved by the ADAM metalloproteinase family (e.g., ADAM10 or ADAM17), releasing a portion of the extracellular fragment and forming a transition intermediate peptide known as “NeXT,” containing the TMD and NICD. γ-secretase, dependent on presenilin, then further cleaves the NeXT peptide, releasing the soluble NICD. Upon translocation to the nucleus, NICD interacts with the transcription factor CBF1 (RBPJ), facilitating the recruitment of co-activator complexes (such as MAML1–3), transforming the “co-repressor complex” into a “co-activator complex”. This complex promotes the transcription of Notch target genes. In the absence of NICD binding, CBF1 recruits co-repressor proteins, thereby downregulating the expression of Notch target genes (Figure 4).

Chaohui Gu et al.^78^ established Notch1-knockout T24 bladder cancer cells and demonstrated that Notch1 deletion significantly impaired sphere-forming ability, indicating a potential role for Notch1 in regulating the self-renewal capacity of BCSCs. Moreover, mutations in NOTCH pathway components such as NOTCH7 gene have been identified as potential immunotherapy biomarkers due to their influence on immune infiltration in the bladder tumor microenvironment.^79^ Bioinformatics analyses have also revealed that NOTCH signaling interacts with immune cell infiltration and EMT pathways, affecting patient prognosis and highlighting its role in tumor microenvironment modulation.^80^ Another study on laminins found that extracellular laminins activate the Notch pathway via integrin α6β4/TRB3/JAG3, thereby inducing tumor cell proliferation or migration in bladder cancer.^59^ Circular RNAs (circRNAs) have been implicated in bladder cancer, and a recent study demonstrated that Circ_0008532 directly interacts with miR-155-5p and miR-330-5p in bladder cancer cell lines and tissues. This interaction mediates the expression of the miR-155-5p/miR-330-5p target gene MTGR1 and activates downstream Notch signaling, thereby promoting tumorigenesis in bladder cancer.^60^

### PI3K/Akt signaling pathway

**Figure 5:**
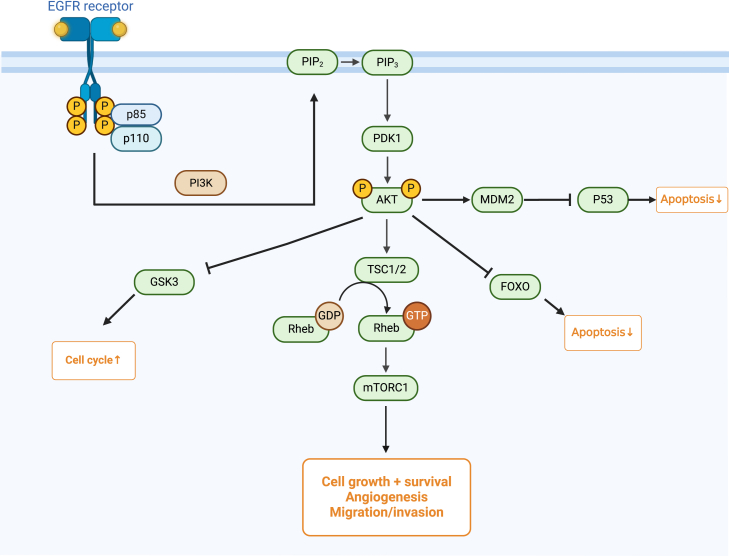
PI3K/Akt signaling pathway The PI3K/Akt signaling pathway regulates cell growth, survival, metabolism, and angiogenesis. Activated by receptor tyrosine kinases (RTKs) upon ligand binding, PI3K generates PIP3, which recruits and activates Akt. Akt phosphorylates key targets like mTOR (promoting protein synthesis), GSK3 (inhibiting apoptosis), and FOXO (suppressing pro-apoptotic genes). PTEN negatively regulates the pathway, preventing excessive cell proliferation. Figure created using BioRender.com.

The PI3K/Akt pathway is implicated in promoting migration and invasion of BCSCs.^81^^,^^82^ The main components of the pathway include:PI3K (Phosphoinositide 3-Kinase): PI3K is composed of a p110 catalytic subunit and a p85 regulatory subunit. These enzymes phosphorylate phosphoinositides, generating docking sites for signaling proteins such as Akt (Figure 5). Upon activation, Akt phosphorylates various downstream targets that regulate cell proliferation, survival, and glucose metabolism. mTOR (mechanistic Target of Rapamycin): As a key downstream effector of Akt, mTOR exists in two complexes: mTORC1 and mTORC2, which control protein synthesis, cell growth, and survival. Akt activation: PIP3 recruits Akt to the plasma membrane, where it is phosphorylated at two key residues (Thr308 by PDK1 and Ser473 by mTORC2), fully activating Akt. Once activated, Akt phosphorylates multiple downstream targets critical for tumorigenesis and cancer progression.^83^ Akt phosphorylates TSC2, inhibiting the TSC1/TSC2 complex, which leads to mTORC1 activation. mTORC1 then promotes protein synthesis and cell growth by phosphorylating S6K1 and 4EBP1. GSK3 (Glycogen Synthase Kinase 3): Akt phosphorylates and inactivates GSK3, which is involved in regulating glycogen synthesis, cell proliferation, and apoptosis. FOXO Transcription Factors: Akt phosphorylates FOXO proteins, resulting in their sequestration and degradation in the cytoplasm, which suppresses the expression of pro-apoptotic genes. MDM2: Akt also phosphorylates MDM2, promoting its translocation to the nucleus, where it binds and degrades the tumor suppressor p53, thereby inhibiting apoptosis.

PI3K inhibitors have demonstrated potent anti-tumor efficacy.^84^ Representative drugs include Pictilisib (GDC-0941), Buparlisib (BKM120), and Pilaralisib (XL147). These inhibitors exhibit broad anti-cancer activity by targeting multiple PI3K isoforms.^65^ Moreover, PI3K inhibitors have shown synergistic anti-BCa effects when combined with STAT3 inhibitors *in vitro*.^84^ The use of the Akt inhibitor MK-2206 effectively alleviated the promotional effects of UPP1 on BLCA tumor growth.^66^ Studies have shown that the natural anticancer agent Jolkinolide B (JB), when used in combination with mTOR inhibitors (such as Temsirolimus, Rapamycin, and Everolimus), exhibits synergistic effects in treating bladder cancer. JB induces dual inhibition of Akt feedback activation and protective autophagy, which enhances the anti-proliferative effects of mTORi in bladder cancer cells.^85^ PI3K/Akt activation also promotes EMT by upregulating integrin-linked kinase, transcription factors (Twist, Snail, Slug) and metalloproteases.^39^

One study demonstrated that the TEAD4 transcription factor, located in the TEA domain, triggers epithelial-mesenchymal transition (EMT) in bladder cancer through the PI3K/Akt pathway. The pathway inhibitor LY294002 blocks TEAD4-induced migration, invasion, and expression of EMT-related markers, proving that TEAD4 is not only an effective prognostic biomarker but also a potential target for treating metastatic BLCA.^61^ Research has shown that the RNA polymerase III subunit G (POLR3G) can activate the PI3K/Akt signaling pathway, promoting EMT in bladder cancer, and enhancing the migration and invasion of bladder cancer cells both *in vitro* and *in vivo*.^86^ Furthermore, Myrtucommulone-A (MC-A) has been found to negatively regulate the EMT process by effectively inhibiting mTOR activity and modulating the PI3K/Akt signaling pathway.^64^ MC-A treatment effectively reduced the phosphorylation of key proteins, demonstrating its inhibitory effects on BCa stem cell proliferation and metastasis.

### Hippo-YAP signaling pathway

**Figure 6:**
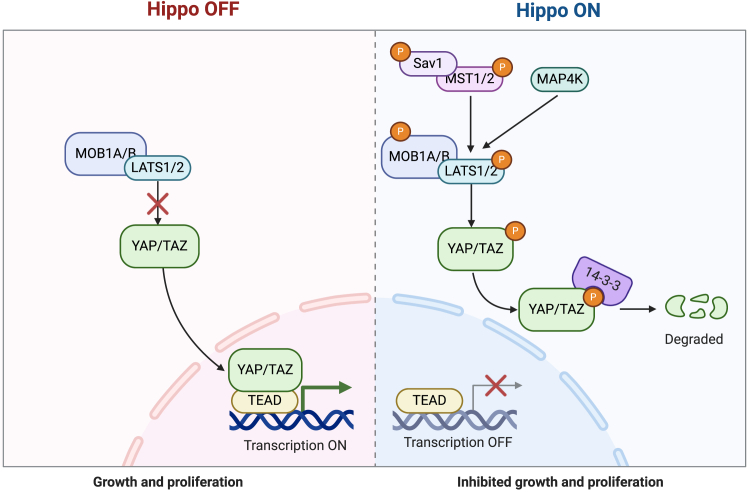
Hippo-YAP signaling pathway Overview of the Hippo-YAP signaling pathway, including the interactions between MST1/2, LATS1/2, YAP/TAZ, and TEAD. When the Hippo pathway is activated, YAP/TAZ are phosphorylated and retained in the cytoplasm, inhibiting abnormal proliferation. In pathway dysregulation, YAP/TAZ translocate to the nucleus, promoting tumor initiation and progression. Figure created using BioRender.com.

The Hippo-YAP signaling pathway consists of two main components: a cytoplasmic kinase cascade and a nuclear transcription module. The cytoplasmic kinase cascade includes MAP4K, MST1/2, and LATS1/2, while the nuclear transcription module is composed of YAP (Yes-associated protein), TAZ (transcriptional coactivator with PDZ-binding motif), and TEAD-1 (TEA domain family member 1).^87^ When the Hippo signaling pathway is activated, MST1/2 phosphorylates LATS1/2, activating them. The activated LATS1/2 then phosphorylate YAP/TAZ, preventing their nuclear translocation. Phosphorylated YAP/TAZ are sequestered in the cytoplasm by 14-3-3 proteins and subsequently degraded via the ubiquitin-proteasome pathway. This process suppresses YAP/TAZ-mediated gene expression, thereby limiting abnormal cell proliferation and tumor formation. In the absence of Hippo pathway activation, unphosphorylated YAP/TAZ translocate into the nucleus, where they bind to TEAD transcription factors. This interaction activates downstream gene expression, promoting cell proliferation, survival, and tissue growth (Figure 6). Recent studies have demonstrated that CSCs harboring mutant p53 can activate WASP-interacting protein (WIP) through upregulation of the PI3K/AKT2 signaling axis, thereby promoting the stabilization of YAP1/TAZ.^88^ In the early stages of bladder cancer, including CIS and NMIBC, genomic alterations induce molecular changes such as dysregulation of the YAP1/JUN-NUAK2-YAP1 positive feedback loop and abnormal activation of the RhoGEF-RhoGTPase-YAP/TAZ cascade. These changes result in excessive YAP/TAZ activation, contributing to tumor initiation and progression.^89^ Progression stage (MIBC). During the progression to MIBC, increased rigidity of the extracellular matrix (ECM) enhances YAP1 activation in bladder cancer cells. The functional regulation of YAP1 involves ubiquitination and deubiquitination mechanisms, with specific regulatory factors playing key roles in these processes.^90^

Inhibiting the YAP-TEAD interaction or reactivating the Hippo kinase cascade could suppress tumor growth and overcome chemotherapy resistance. Previous research demonstrated that metformin, a classic antidiabetic drug, effectively inhibits bladder cancer cell proliferation both *in vitro* and *in vivo*^91^ and can inhibit bladder cancer progression by suppressing the reprogramming of BCSCs.^92^ Studies have suggested that metformin targets the YAP1-TEAD4 complex via AMPKα, regulating the expression of CCNE1/2 (Cyclin E) in bladder cancer cells, inducing cell-cycle arrest and ultimately affecting cell growth.^93^ Additionally, a study screening a small-molecule compound library to identify novel drugs targeting the Hippo pathway found that DMPCA (N-(3,4-dimethoxyphenethyl)-6-methyl-2,3,4,9-tetrahydro-1H-carbazol-1-amine) can induce phosphorylation of LATS1 and YAP1/TAZ in bladder cancer cells. Both *in vitro* and *in vivo* experiments confirmed that DMPCA effectively inhibits bladder cancer cell growth.^94^

YAP is essential for maintaining the stemness and proliferation of BCSCs and is associated with the expression of markers such as OV6 and ALDH1.^13^^,^^95^ YAP activates the transcription and translation of PDGFB through TEAD-1, producing PDGF-BB (platelet-derived growth factor subunit B), which prevents YAP from being phosphorylated by LATS1/2 and increases its nuclear localization.^13^ In ALDH1+ BCSC cells, YAP activity is also evident, and silencing YAP reduces the expression of the ALDH1A1 gene.^95^ Moreover, YAP maintains CSC characteristics by inducing autophagy, which is crucial for CSC survival, stemness maintenance, and growth. Autophagy also facilitates CSC invasion and metastasis, induces dormancy, and helps CSCs develop resistance to chemotherapy and radiotherapy, thereby improving their survival rate. Combining the YAP1 inhibitor vitipofen with the COX2 inhibitor celecoxib in chemotherapy can reduce BCSC characteristics and enhance chemotherapy efficacy.^67^ YAP1 also directly induces SOX2 expression by binding to enhancer regions, promoting the expansion and maintenance of urothelial CSCs, thereby contributing to their drug resistance.

### STAT3 signaling pathway

**Figure 7:**
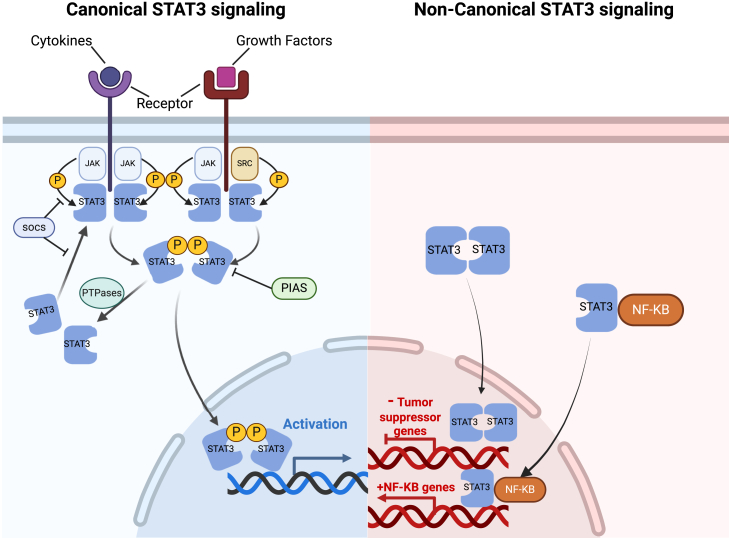
STAT3 signaling pathway Overview of the STAT3 signaling pathway, highlighting its activation by JAK and SRC kinases through classical and non-classical mechanisms. It shows the critical role of STAT3 in gene transcription, cell survival, and its involvement in oncogenic signaling networks associated with cancer progression. Figure created using BioRender.com.

Activated STAT3 drives expression of pluripotency factors (e.g., Oct4 and Nanog) and adhesion molecules (CD44) in bladder CSCs, promoting self-renewal, invasion and tumorigenicity. JAK kinases activate STAT3 by binding to cell surface receptors, while SRC kinases, as non-receptor tyrosine kinases, aslo activate STAT3 through a classical pathway. The activation process involves cytokines (such as IL-6) binding to receptors, which leads to JAK kinase-mediated phosphorylation of the receptor and recruitment of STAT3. Phosphorylation of STAT3 at Tyr705 triggers its dimerization, allowing it to translocate into the nucleus and initiate gene transcription. In the non-classical pathway, unphosphorylated STAT3 can also influence gene transcription and interact with NF-κB to modulate each other’s activity. STAT3 activity is regulated by suppressors such as SOCS, PIAS, and PTPases. Beyond regulating gene expression, STAT3 also participates in non-transcriptional activities, including interactions with the cytoskeleton. As an essential intracellular signaling node, STAT3 integrates signals from key pathways such as EGFR, RAS-RAF, C-MET, and TGF-β, thereby forming a complex oncogenic signaling network (Figure 7).^96^^,^^97^ Ho et al.^98^ reported that Stat3-transgenic mice developed invasive bladder cancer directly from CIS, bypassing the non-invasive papillary tumor stage. While direct mutations in the STAT3 gene itself are relatively rare, alterations in genes like FGFR3, a frequently mutated gene in bladder cancer, can indirectly lead to STAT3 activation.

A study on the STAT3 inhibitor WP1066 demonstrated that when administered at varying doses to bladder cancer cells, WP1066 could block STAT3 phosphorylation, reduce cancer cell survival and proliferation, and induce apoptosis.^68^^,^^99^ Several natural products have also been identified as potential inhibitors targeting the STAT3 pathway in bladder cancer. Paeoniflorin (Pae), the major component of Radix Paeoniae Alba (RPA), was shown to inhibit the proliferation of human BCa cell lines in a concentration- and time-dependent manner.^100^ Pae, in combination with cisplatin (Cis), synergistically inhibits tumor growth. Pae treatment also alleviates the body weight loss induced by Cis. Furthermore, Pae not only induces apoptosis in cancer cells but also inhibits the nuclear translocation of STAT3 in bladder cancer cells, thereby suppressing the proliferation of human BCa cell lines in a concentration- and time-dependent manner.^69^ According to an experiment, δ-tocotrienol (δ-T3), one of the vitamin E isomers, demonstrated the strongest cytotoxic activity against human bladder cancer cells compared to other vitamin E isomers. δ-T3 induced BC cell apoptosis by reducing the levels of STAT3 protein in the nuclear fraction and its transcriptional activity. Additionally, low-dose δ-T3 enhanced the cytotoxic effects of gemcitabine on human bladder cancer cells, increasing their sensitivity to chemotherapy. Increasingly, studies targeting the STAT3 pathway for therapy are emerging. The overexpression of the mitotic kinase BUB1 has been implicated in the initiation and progression of various cancers, including bladder cancer, by mediating the STAT3 signaling pathway.^70^ CircRPPH1, a non-coding RNA exhibiting tissue-specific expression patterns, accelerates bladder cancer proliferation and migration by upregulating the STAT3 signaling pathway.^101^ EZH2, a member of the polycomb group of proteins, plays a crucial role in embryonic stem cell pluripotency and self-renewal.^31^ Experimental results suggest that EZH2 may promote the proliferation and migration of bladder cancer cells through the STAT3 pathway, highlighting its potential role in bladder cancer progression.^102^ These findings suggest that downregulating or blocking the STAT3 pathway could be a promising therapeutic strategy to prevent bladder cancer progression. Moreover, fractionated irradiation promotes the activation of the CSC-related STAT3 signaling pathway in BC cells, with surviving cells exhibiting enhanced migration, invasion, and CSC-like characteristics. Knockdown of STAT3 expression or inhibition of STAT3 activity significantly reduces the self-renewal capacity and tumorigenicity of radioresistant BC cells.^103^ Furthermore, Sun et al.^104^ found that inhibition of STAT signaling, either through genetic approaches or treatment with diindolylmethane, reduced the invasiveness of bladder cancer cells, highlighting the potential of targeting STAT pathways in therapeutic strategies.

## Drug resistant mechanism of BCSCs

### ABC transport proteins

The drug resistance of BCSCs is closely linked to the function of ATP-binding cassette (ABC) transporters, a family of membrane-bound proteins that actively pump out metabolites, drugs, and toxins from cells. This action significantly reduces intracellular drug concentrations, contributing to the resistance of tumor cells.^105^ In BCSCs, the elevated expression of several ABC transporter family members, such as ABCB1, ABCG2, and ABCC1, is a key factor driving resistance. These proteins not only expel conventional chemotherapeutic agents like docetaxel and cisplatin but also efflux various signaling molecules, including lysophosphatidylinositol and interleukin-1β, which provides CSCs with a survival advantage.^106^

Research indicates that ABC transporters play a dual role in BCSC drug resistance: they directly pump out chemotherapeutic drugs and are also involved in inflammatory signaling and the maintenance of stemness. For instance, ABCB5 regulates IL-8-dependent signaling pathways to preserve stem cell properties in melanoma, while ABCG2 enhances the tumorigenic potential of CSCs. These findings suggest that ABC transporters contribute to drug resistance through multiple mechanisms in BCSCs.^107^

However, targeting ABC transporters for therapeutic purposes presents significant challenges. On the one hand, BCSCs typically express multiple ABC transporters simultaneously, and inhibiting a single transporter (e.g., ABCB1) may be counterbalanced by the compensatory activity of other transporters. On the other hand, the regulatory pathways governing the expression of ABC transporters, such as Wnt, Notch, and NRF2, are not fully understood, which limits the development of effective targeted therapies.^108^

### Lineage plasticity

Tumor cells evade targeted therapies through lineage plasticity, which offers a critical survival advantage for heterogeneous tumors. This plasticity enhances tumor growth potential, helps escape immune surveillance, enables the development of both innate and acquired resistance, and increases metastatic capabilities. Lineage plasticity has emerged as one of the central mechanisms by which tumors develop resistance to targeted therapies, demonstrating the dynamic adaptability of tumor cells. This ability allows them to circumvent therapies directed at specific molecules or phenotypic markers by transitioning between different lineage states. Particularly in epithelial-derived cancers, tumor cells can undergo epithelial-to-mesenchymal transition (EMT) to adopt a mesenchymal-like phenotype.^7^ This shift is often linked with resistance to therapies targeting epithelial markers, as mesenchymal-like cells exhibit significantly reduced reliance on certain targeted pathways, complicating treatment efforts.^8^

The manifestation of lineage plasticity in malignant tumors is particularly pronounced. Tumor cells continuously adapt and evolve across both temporal and spatial dimensions, which complicates and reduces the efficacy of targeted therapies. Furthermore, cancer cells can acquire stem cell-like traits, including high plasticity and self-renewal capacity. By generating drug-resistant cell populations, they further strengthen their resistance to treatment.

External stimuli, including targeted therapies, can reprogram the gene expression profiles of tumor cells, inducing dynamic changes in their lineage states. This process is largely governed by epigenetic mechanisms, such as DNA methylation and histone modifications, the action of non-coding RNAs (such as miRNA and lncRNA), and the regulation of key transcription factors like SOX2, ZEB1, and MYC. These factors together contribute to the shifting treatment dependencies of tumor cells.^109^ For example, tumor cells may transition from relying on hormone receptor signaling to utilizing alternative pathways such as PI3K/AKT, Wnt, or Notch for survival. This transcriptional reprogramming not only undermines the effectiveness of targeted therapies but also complicates second-line treatment strategies.

### Apoptotic resistance

BCSCs evade apoptosis through multiple mechanisms, which involve both extrinsic and intrinsic pathways. These pathways are mediated by death receptor signals and mitochondrial signals, respectively, but BCSCs suppress the activation of both pathways by regulating key molecules. A particularly notable feature in BCSCs is the high expression of anti-apoptotic proteins from the Bcl-2 family (such as BCL2, MCL1, and BCL-XL). These proteins prevent the release of pro-apoptotic factors, like cytochrome *c*, by inhibiting mitochondrial outer membrane permeability (MOMP), thus blocking intrinsic apoptosis. In addition, BCSCs often exhibit downregulation of pro-apoptotic factors (such as Bax and Bak) and inhibition of caspase activity, which further diminishes the apoptotic effects induced by treatments. The dynamic regulation of these anti-apoptotic genes enables BCSCs to survive under the pressure of chemotherapy and radiotherapy, and to re-enter the cell cycle and proliferate once treatment ceases. Urothelial CSCs express high levels of IL11, IL18, and IL23 mRNA. These interleukins enhance tumor cell survival and growth *in vitro* by activating anti-apoptotic genes, including cFLIP/FLAME-1 and Bcl-xL.^105^

Furthermore, BCSCs increase their drug resistance by inhibiting the extrinsic apoptosis pathway. The expression of death receptors (such as Fas) and their ligands (FasL) is significantly reduced in BCSCs, while upregulation of anti-apoptotic molecules (such as FLIP protein) blocks death receptor-mediated signaling. This regulation directly weakens the extrinsic apoptotic signals triggered by treatment. In addition, the abnormal activation of key signaling pathways synergizes with the evasion of apoptosis. For example, the overactivation of the PI3K/AKT signaling pathway in BCSCs not only suppresses pro-apoptotic factor functions but also enhances the expression of anti-apoptotic genes. Similarly, the abnormal activation of the Wnt/β-catenin pathway further supports BCSC survival by regulating stemness genes. The interaction of these pathways contributes to the higher resistance of BCSCs to targeted therapies.^110^

The tumor microenvironment also plays a crucial role in supporting BCSC apoptosis evasion. Immunosuppressive cells and fibroblasts in the microenvironment secrete cytokines such as IL-6 and TGF-β, which activate the STAT3 and SMAD signaling pathways, thereby enhancing the anti-apoptotic capacity of BCSCs. Additionally, under hypoxic conditions, the upregulation of HIF-1α further promotes the expression of anti-apoptotic genes and reduces BCSC sensitivity to treatment. External stimuli can also induce epigenetic modifications (such as DNA methylation and histone modification) to downregulate pro-apoptotic genes (e.g., PUMA and NOXA), while the downregulation of non-coding RNAs (such as miR-34a) disrupts the p53-dependent apoptosis pathway. The combined effects of these internal and external factors create a complex regulatory network that allows BCSCs to evade apoptosis in response to various therapeutic interventions.^111^

### Quiescence and cell cycle regulation

Quiescence and cell cycle regulation are key mechanisms underlying drug resistance in CSCs. In the quiescent state, CSCs enter a non-dividing phase (G0 phase), exhibiting low metabolic and proliferative activity, which allows them to evade traditional therapies that target rapidly dividing cells.^110^ Research on CSCs has shown that cyclin-dependent kinase inhibitors, such as P21 and P53, suppress the expression of Cyclin D1, leading to cell-cycle arrest at the G0/G1 phase. This results in a general insensitivity to chemotherapy, often contributing to tumor recurrence. Quiescence serves as a dynamic and reversible survival strategy, enabling CSCs to persist under therapeutic stress and later re-enter the cell cycle once environmental conditions improve or treatment ceases, which facilitates tumor relapse.^107^

Chemotherapeutic agents typically kill cancer cells by disrupting cell division, but CSCs in the quiescent state are naturally resistant to these drugs because they are not actively dividing. Similarly, radiation therapy induces DNA damage to kill cancer cells, but quiescent CSCs are less sensitive to such damage and possess enhanced DNA repair capabilities, such as the upregulation of p53-mediated repair pathways, further contributing to their resistance. Targeted therapies also face challenges, as many drugs that target signaling pathways require active signals in proliferating cells. However, quiescent CSCs exhibit lower metabolic and signaling activity, making them difficult to target effectively in treatment.

Quiescence is regulated by several key signaling pathways, including the Notch, Wnt/β-catenin, and Hedgehog pathways, which are crucial for maintaining stemness and regulating the quiescent state. Additionally, cell cycle inhibitors such as p21 and p27 promote entry into the G0 phase by inhibiting cyclin-dependent kinases (CDKs). Metabolic regulation is another important characteristic of the quiescent state, as CSCs show reduced oxygen consumption and reliance on glycolysis, which further enhances their drug resistance.^112^

### Epigenetics

Epigenetic modifications, such as DNA methylation, histone modifications, and regulation by non-coding RNAs, play pivotal roles in promoting drug resistance in CSCs and facilitating tumor recurrence by regulating gene expression, stem cell phenotypes, and alterations in the tumor microenvironment. For instance, upregulation of histone deacetylases (HDACs) can activate genes associated with drug resistance, thus promoting cancer cell survival and recurrence. DNA methylation, in particular, is central to the development of drug resistance in CSCs. Under normal conditions, the promoter regions of tumor suppressor genes like p16 and p53 are methylated, leading to silencing of their expression and allowing tumor cells to evade growth suppression.^113^ However, certain subpopulations of tumor cells can develop reversible drug resistance upon initial exposure to chemotherapy (and possibly other stressors), thereby enabling these cells to survive and protect the larger tumor population from extinction due to lethal drug exposure.^114^ Studies indicate that during cancer progression, especially under chemotherapy-induced stress, a hypomethylated state can induce aberrant expression of critical genes, activate oncogenic pathways, and enhance stem cell properties. In particular, during the early stages of tumorigenesis, DNA hypomethylation promotes the formation and survival of CSCs, a process closely linked to the activation of the PI3K-AKT signaling pathway. E2F3, a cell cycle regulator associated with aggressive bladder cancer, also plays a role. CSC-like cells show increased accessibility of the E2F3 promoter and elevated E2F3 expression, which drive cell migration, invasiveness, and drug resistance. Treatment with DNA methylation inhibitors can block the transition to a CSC-like state and reduce E2F3 expression.^115^

Additionally, non-coding RNAs, particularly microRNAs (miRNAs) and long non-coding RNAs (lncRNAs), are crucial in modulating drug resistance in breast cancer stem cells (BCSCs). Research has shown that miRNAs regulate BCSC drug resistance by influencing processes such as cell cycle progression, apoptosis, and drug metabolism. For example, miR-21 has been shown to promote drug resistance in BCSCs by downregulating tumor suppressor genes. Similarly, lncRNAs such as MALAT1 enhance BCSC resistance to chemotherapy by regulating the expression of ABC transporters, thereby altering extracellular drug concentrations.^116^

## Conclusion and future prospects

CSCs possess remarkable heterogeneity and plasticity,^117^ enabling them to survive conventional chemotherapy and immunotherapy. Although several FDA approved agents are under investigation for CSC suppression, durable clinical responses remain limited. To overcome treatment resistance and improve outcomes, development of novel molecular therapies targeting CSC specific pathways is essential. Comprehensive analyses have demonstrated that CSC populations vary widely across cancer types and evolve under chemotherapeutic pressure, underscoring the need to elucidate the key signaling networks that drive their self-renewal and tumorigenic potential.

In bladder cancer, future research must prioritize the identification and validation of specific biomarkers to distinguish and isolate BCSC subtypes. Such markers will enable precision stratification and facilitate the design of BCSC directed therapies. Integrating BCSC targeted agents with chemotherapy, immunotherapy, and strategies that remodel the tumor microenvironment holds promise for overcoming relapse and resistance. Finally, leveraging advances in single cell genomics, gene editing, and robust *in vitro* and *in vivo* models will accelerate translation of BCSC biology into personalized clinical interventions. Insights gained from BCSC studies not only promise breakthroughs in early detection, targeted treatment, and prognosis of bladder cancer but also provide a paradigm for addressing CSC driven therapy in other malignancies.

## Acknowledgments

The author(s) declare that financial support was received for the research, authorship, and/or publication of this article. This research has been supported by the Central Guidance on the Local Science and Technology Development Fund of Shanxi Province (No. YDZJSX2021C010), the Patent Conversion Project Fund of Shanxi Province (No. 202304008).

## Author contributions

Conceptualization, investigation, writing – original draft, writing – review and editing, methodology, S.L.; conceptualization, writing – original draft, writing – review and editing, visualization, K.Y.; writing – original draft, writing – review and editing, Z.J.; formal analysis, writing – original draft, P.Y.; conceptualization, writing – review and editing, Y.W.; formal analysis, writing – original draft, J.L.; writing – review and editing, M.X.; writing – review and editing, X.G.; writing – original draft, Q.X.; writing – formal analysis, H.Z.; writing – original draft, Q.L.; conceptualization, investigation, writing – original draft, writing – review and editing, C.L.; conceptualization, funding acquisition, project administration, resources, writing – review and editing, X.Y.

## Declaration of interests

The authors declare that they have no known competing financial interests or personal relationships that could have appeared to influence the work reported in this paper.
